# Development and validation of a risk prediction model for postoperative pneumonia in elderly non-cardiac surgery patients: a retrospective cohort study

**DOI:** 10.1038/s41598-025-20584-8

**Published:** 2025-10-21

**Authors:** Xuhui Cong, Xuli Zou, LuLu Jiang, Ruilou Zhu, Yubao Li, Lu Liu, Jiaqiang Zhang

**Affiliations:** 1 Department of Anesthesia and Perioperative Medicine, Zhengzhou University People’s Hospital and Henan Provincial People’s Hospital, Zhengzhou, Henan People’s Republic of China; 2 Xinxiang Medical University, Xinxiang, Henan People’s Republic of China; 3 Zhengzhou University, Zhengzhou, Henan People’s Republic of China

**Keywords:** Postoperative pneumonia, Predictive model, Perioperative management, Risk assessment, Noncardiac surgery, Metabolic disorders, Respiratory tract diseases

## Abstract

Postoperative pneumonia (POP) is a prevalent, severe complication in elderly noncardiac surgical patients, linked to extended hospital stays, increased healthcare costs, and higher mortality. Existing predictive models are often limited by single-center data, small cohorts, or restricted variables, highlighting the need for a comprehensive tool integrating multi-phase perioperative factors. This retrospective study analyzed 44,740 patients aged ≥ 65 years who underwent noncardiac surgery (November 2014–April 2022) at Henan Provincial People’s Hospital, with 3187 (7.1%) developing POP. Patients were stratified into development (n = 31,320) and validation (n = 13,420) cohorts via 70:30 random split. Key predictors were identified using LASSO logistic regression (from 44 candidates), followed by multivariate logistic regression with forward stepwise selection. Model performance was evaluated via AUC (discrimination), calibration (Hosmer–Lemeshow test, Brier score), clinical utility (decision curve analysis [DCA]), and interpretability (SHAP analysis). The final model included 9 predictors: anesthesia duration, anesthesia type, smoking status, pulmonary disease history, intraoperative colloid volume, preoperative anticoagulant/antihypertensive/steroid use, and intraoperative sufentanil dose. It demonstrated strong discrimination (validation AUC = 0.804, 95% CI 0.790–0.818) and good calibration (development: Hosmer–Lemeshow χ^2^ = 5.45, *P* = 0.79; validation: χ^2^ = 7.81, *P* = 0.55; Brier score = 0.058 for both). A derived nomogram (optimal cutoff = 190) showed high sensitivity (76.3%) and specificity (69.6%). DCA confirmed net benefit across 0–89% (development) and 0–88% (validation) thresholds. SHAP analysis identified prolonged anesthesia and pulmonary disease history as top predictors. This multifactorial model reliably predicts postoperative pneumonia in elderly noncardiac surgical patients using routinely collected perioperative data, with good discrimination and calibration. By integrating a wider range of variables than prior models, it enhances predictive accuracy and clinical applicability. External validation in multicenter prospective cohorts is needed to confirm its generalizability and support clinical integration.

## Introduction

Postoperative pneumonia (POP) ranks among the most frequent and serious complications following noncardiac surgery—especially in elderly patients and those with multiple comorbidities. Moreover, POP not only extends hospital stays and drives up healthcare costs but also markedly increases postoperative mortality risk, thereby undermining both short-term recovery and long-term outcomes^1,2^.

However, most existing studies examine POP in isolation—either by targeting specific risk factors or focusing on narrow surgical subgroups. Consequently, a comprehensive, validated model that incorporates variables from the preoperative, intraoperative, and postoperative phases remains lacking^3,4^. Recent studies have identified advanced age, hypoalbuminemia, prolonged surgical duration, and ICU admission as independent predictors of postoperative pancreatic fistula (POP) ^5,6^. However, most existing predictive models have significant limitations. They are often developed using single-center data or small patient cohorts, which restricts their generalizability, and they fail to account for the complex interactions between multiple perioperative variables^3,4^.

Given the growing emphasis on precision medicine and perioperative optimization—coupled with evidence from systematic reviews supporting POP preventive strategies like perioperative oral care^7^—a robust, generalizable, and clinically applicable risk prediction model for POP is urgently needed to guide individualized decision-making. This study aims to address this gap by developing and validating a multifactorial predictive model using a large retrospective cohort. By integrating diverse perioperative variables—including patient demographics, surgical factors, and postoperative management data—it seeks to provide an evidence-based tool that helps clinicians identify high-risk patients and tailor perioperative interventions accordingly.

Despite increasing recognition of postoperative pneumonia as a major cause of morbidity and mortality in elderly noncardiac surgical patients, existing prediction models are limited by single-center cohorts, narrow surgical populations, and inadequate incorporation of intraoperative variables. These shortcomings restrict their generalizability and clinical applicability. To address this gap, the present study aimed to develop and internally validate a comprehensive risk prediction model for POP using a large retrospective cohort. By integrating preoperative, intraoperative, and perioperative variables, this study sought to establish a clinically applicable tool to enable early identification of high-risk patients and guide targeted preventive strategies.

## Methods

### Data source and preprocessing

Electronic medical records of noncardiac surgical patients aged ≥ 65 years treated at Henan Provincial People’s Hospital between November 2014 and April 2022 were retrospectively analyzed. This study was approved by the Medical Ethics Committee of Henan Provincial People’s Hospital under the project title “Multicenter Retrospective Study on Anesthesia-related Risk Factors for Perioperative Mortality in Elderly Non-Cardiac Surgery Patients.” The approval number is (2021) Ethics Review No. 157, dated November 16, 2022. The requirement for written informed consent was waived due to the retrospective design, in accordance with the Declaration of Helsinki and relevant national regulations. Demographic, comorbidity, and laboratory data were extracted in compliance with all applicable ethical standards.

All data were extracted from the hospital’s Health Information System (HIS) and organized into a perioperative clinical data warehouse established by Hangzhou Le9 Healthcare Technology Co., Ltd. This structured database integrates demographic information, surgical and anesthesia records, medication history, laboratory results, and postoperative outcomes. A standardized data extraction protocol was used to ensure accuracy and completeness. After extraction, data were cleaned, preprocessed, and analyzed for model development and internal validation. The database reflects real-world perioperative clinical practice in a high-volume tertiary hospital, providing a comprehensive foundation for large-scale risk modeling.

### Study population

In this retrospective cohort, patients were included if they:

1. Underwent noncardiac surgery with standard general anesthesia or general anesthesia combined with nerve block (including abdominal, ENT, orthopedic, gynecologic, neurosurgical, ophthalmic, thoracic, stomatologic, and urologic procedures);
2. Were aged ≥ 65 years;
3. Had an ASA physical status classification of I–III;
4. Had no preoperative pneumonia or other acute respiratory infections.

The exclusion criteria were:

1. ASA classification of IV or above;
2. Cardiac or major vascular surgical procedures;
3. Admission for infectious diseases;
4. Diagnosis with pneumonia or severe respiratory infection preoperatively;
5. Critically ill ICU patients;
6. Missing data > 20% of predefined key perioperative variables.

**Fig. 1:**
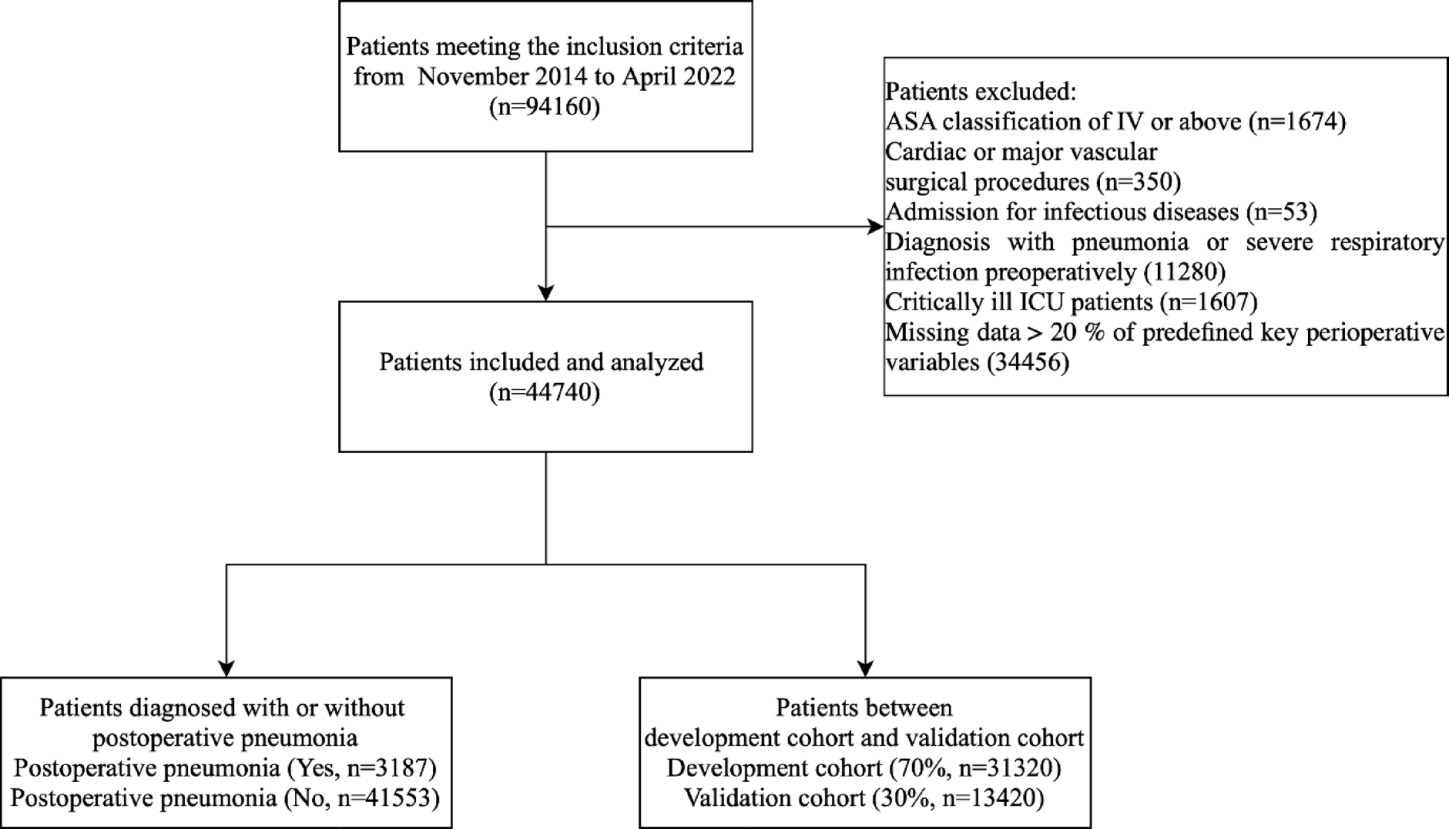
Patient flow diagram. ASA, American society of anesthesiologists.

After applying these criteria, 44,740 eligible surgical cases were included, with 3187 developing postoperative pneumonia (POP) and 41,553 not. The cohort was randomly divided into a development cohort (n = 31,320) and a validation cohort (n = 13,420) using a fixed random seed and a 70:30 split ratio. This stratified division ensured proportional representation of POP and non-POP cases in both subsets. The patient selection and data allocation process is illustrated in Fig. 1.

### Clinical variables and definitions

Perioperative data were collected for each patient spanning from 7 days before surgery to 7 days after surgery. Demographic characteristics included sex, age, height, weight, and ASA physical status classification. Surgery-related variables comprised surgical duration, anesthesia duration, type of anesthesia, emergency surgery status, intraoperative blood loss, and the volume of crystalloid and colloid fluids administered.

Information on comorbidities and past medical history included prior lung infections, smoking history, hypertension, diabetes mellitus, hyperlipidemia, and chronic pulmonary disease. Preoperative laboratory parameters consisted of red blood cell count, lymphocyte count, basophil count, platelet count, total bilirubin, serum albumin, glucose, white blood cell count, and hemoglobin levels.

Data on preoperative medication use covered glucocorticoids, anticoagulants, antidiabetic drugs, antihypertensive agents, and statins, including routine steroid use and prior anticoagulation. Intraoperative medications included opioid analgesics, opioid antagonists, non-opioid analgesics and adjuvants, local anesthetics, inhalational agents, sedatives, and antibiotics. Intraoperative support measures such as nasogastric tube placement and blood transfusion were also recorded. Postoperative medications included the use of analgesics during the immediate recovery period.

*Definition of POP* Postoperative pneumonia was defined as new or progressive pulmonary infiltrates on chest imaging (X-ray or CT) within 48 h of surgery, accompanied by at least one clinical symptom (fever, productive cough, leukocytosis), with confirmation via laboratory or microbiological testing when necessary.

The 48-h timeframe was chosen to capture pneumonia most directly linked to intraoperative factors and the immediate perioperative course. This aligns with postoperative surveillance protocols in many surgical settings and has been used in prior studies to assess early pulmonary complications^8,9^. Wang et al. (2023) highlighted the value of early postoperative windows (particularly within 48 h) for developing reliable prediction models of bacterial pneumonia after cardiovascular surgery^10^.

Notably, this criterion is more restrictive than typical clinical practice, which may include pneumonia diagnosed up to 72 h or later. While this may underestimate POP incidence by excluding later-onset but clinically relevant cases, the stricter definition enhances diagnostic specificity and focuses the model on early, potentially preventable events tied to operative care.

### Statistical analysis

Continuous variables are reported as medians with interquartile ranges (IQR) and compared using the Mann–Whitney U test. Categorical variables are presented as frequencies and percentages, with comparisons using the chi-square test or Fisher’s exact test, as appropriate.

Missing data were addressed using Multiple Imputation by Chained Equations (MICE), which generates multiple imputed datasets by iteratively estimating missing values based on observed variable relationships. This method more accurately represents missing data than simpler approaches (e.g., mean/median imputation).

For variable selection, least absolute shrinkage and selection operator (LASSO) logistic regression (via the glmnet package in R) was used to identify key predictors. LASSO performs penalized regression to reduce model complexity and prevent overfitting, focusing on the most influential variables. These variables were then included in a multivariable logistic regression model using forward stepwise selection to minimize the Akaike Information Criterion (AIC), ensuring model parsimony.

Verification of logistic regression assumptions:

1. *Linearity in the logit scale* For continuous predictors (e.g., anesthesia duration, intraoperative colloid volume), the Box-Tidwell test assessed linearity with the logit-transformed outcome (POP). No significant departures from linearity were observed (all *P* > 0.05), confirming adherence.
2. *Independence of observations* Given the retrospective cohort design, patients were treated as independent (no overlapping surgical episodes or shared perioperative teams), with no evidence of clustering.
3. *Absence of multicollinearity* A correlation matrix and variance inflation factors (VIF)/tolerance values quantified inter-variable relationships. All predictors had VIF < 10 and tolerance > 0.1 (Table 3), indicating acceptable collinearity.
4. *Adequate sample size* The ratio of events (POP cases, n = 3187) to predictors (n = 9) exceeded 30:1, well above the recommended 10:1 threshold for stable logistic regression coefficients.

No substantial assumption violations were identified, so no corrective measures (e.g., variable transformation) were needed.

Odds ratios (ORs) with 95% confidence intervals (CIs) were calculated to quantify the strength and precision of associations.

Model discrimination was evaluated using the receiver operating characteristic (ROC) curve, with the area under the curve (AUC) quantifying performance. Calibration was assessed via calibration plots and Spiegelhalter’s Z test, where non-significant results indicated good agreement between predicted and observed outcomes.

A nomogram derived from the final logistic regression model visually represents variable contributions and estimates individual POP risk, providing clinicians with a point-based scoring system for patient-specific prediction.

To enhance model interpretability, SHAP (SHapley Additive exPlanations) analysis was conducted using the shap package in R. Derived from game theory, SHAP values quantify each feature’s contribution to individual predictions by measuring its deviation from the model’s baseline (average prediction).

All analyses were performed in R version 4.3.1, with visualizations (ROC curves, calibration plots, nomogram) generated using the rms package. All tests were two-sided, with *P* < 0.05 considered statistically significant.

## Results

### Patient characteristics and cohort description

A total of 44,740 patients who underwent noncardiac surgery—including general, orthopedic, urological, gynecological, and noncardiac thoracic procedures—were included in the final cohort. Of these, 3187 (7.1%) developed postoperative pneumonia (POP).

The cohort was randomly split into a development cohort (n = 31,320) and a validation cohort (n = 13,420) at a 70:30 ratio. Among patients with POP, 2228 were in the development cohort and 959 in the validation cohort.

Baseline characteristics and perioperative variables for patients with and without POP are summarized in Table 1; differences between the development and validation cohorts are shown in Table 2. Key variables were similarly distributed across cohorts, supporting model consistency.

**Table 1:** Baseline demographic and clinical characteristics of included patients with or without postoperative pneumonia.

		Postoperative pneumonia		
Variables	Total (n = 44,740)	No (n = 41,553)	Yes (n = 3187)	*P*
Sex, n (%)				< 0.01
Male	23,503 (53)	21,638 (52)	1865 (59)	
Female	21,237 (47)	19,915 (48)	1322 (41)	
Age, (median [Q1, Q3]), yr	70 (67, 74)	70 (67, 74)	70 (67, 74)	< 0.01
Height, (median [Q1, Q3]), cm	163.67 (157.5, 168.16)	163.45 (157.52, 168.15)	165.2 (157.23, 168.28)	0.18
Weight, (median [Q1, Q3]), kg	64.85 (56.5, 70)	64.85 (56.83, 70)	64.8 (56, 70)	0.17
ASA classification, n (%)				< 0.01
ASA I	518 (1)	515 (1)	3 (0)	
ASA II	31,872 (71)	29,340 (71)	2532 (79)	
ASA III	12,350 (28)	11,698 (28)	652 (20)	
Duration of surgery, (median [Q1, Q3]), minute	130 (75, 210)	125 (70, 200)	200 (140, 275)	< 0.01
Duration of anesthesia, (median [Q1, Q3]), minute	145 (85, 225)	140 (84, 220)	220 (160, 295)	< 0.01
Anesthesia type, n (%)				< 0.01
Simple general anesthesia	31,468 (70)	30,302 (73)	1166 (37)	
General anesthesia combined with nerve block	13,272 (30)	11,251 (27)	2021 (63)	
Emergency surgery, n (%)				0.31
No	39,486 (88)	36,655 (88)	2831 (89)	
Yes	5254 (12)	4898 (12)	356 (11)	
Surgery type, n (%)				< 0.001
Abdominal_surgery	12,941 (29)	11,363 (27)	1578 (50)	
ENT	2326 (5)	2237 (5)	89 (3)	
General_surgery	1827 (4)	1771 (4)	56 (2)	
Gynecologic_surgery	2110 (5)	2031 (5)	79 (2)	
Neurosurgery	2825 (6)	2701 (7)	124 (4)	
Ophthalmic_surgery	2910 (7)	2866 (7)	44 (1)	
Orthopedic_surgery	6445 (14)	6031 (15)	414 (13)	
Other_surgeries	2828 (6)	2660 (6)	168 (5)	
Stomatology	1163 (3)	1109 (3)	54 (2)	
Thoracic_surgery	1810 (4)	1482 (4)	328 (10)	
Urology	7555 (17)	7302 (18)	253 (8)	
Smoking status, n (%)				< 0.01
No	34,002 (76)	32,063 (77)	1939 (61)	
Yes	10,738 (24)	9490 (23)	1248 (39)	
Hypertension, n (%)				0.21
No	35,445 (79)	32,892 (79)	2553 (80)	
Yes	9295 (21)	8661 (21)	634 (20)	
Diabetes mellitus, n (%)				< 0.01
No	39,713 (89)	36,956 (89)	2757 (87)	
Yes	5027 (11)	4597 (11)	430 (13)	
Hyperlipidemia, n (%)				0.99
No	44,579 (100)	41,404 (100)	3175 (100)	
Yes	161 (0)	149 (0)	12 (0)	
History of pulmonary disease, n (%)				< 0.01
No	43,774 (98)	40,891 (98)	2883 (90)	
Yes	966 (2)	662 (2)	304 (10)	
Preoperative red blood cell abnormalities, n (%)				0.44
Low	17,432 (39)	16,157 (39)	1275 (40)	
Normal	27,019 (60)	25,126 (60)	1893 (59)	
High	289 (1)	270 (1)	19 (1)	
Preoperative lymphocyte abnormalities, n (%)				0.06
Low	8350 (19)	7706 (19)	644 (20)	
Normal	35,492 (79)	33,008 (79)	2484 (78)	
High	898 (2)	839 (2)	59 (2)	
Preoperative basophil abnormalities, n (%)				< 0.01
Normal	42,994 (96)	39,969 (96)	3025 (95)	
High	1746 (4)	1584 (4)	162 (5)	
Preoperative platelet count abnormalities, n (%)				0.15
Low	2755 (6)	2540 (6)	215 (7)	
Normal	39,937 (89)	37,125 (89)	2812 (88)	
High	2048 (5)	1888 (5)	160 (5)	
Preoperative total bilirubin, n (%)				0.85
Low	1713 (4)	1587 (4)	126 (4)	
Normal	38,990 (87)	36,223 (87)	2767 (87)	
High	4037 (9)	3743 (9)	294 (9)	
Preoperative albumin abnormalities, n (%)				< 0.01
Low	22,319 (50)	20,662 (50)	1657 (52)	
Normal	22,419 (50)	20,890 (50)	1529 (48)	
High	2 (0)	1 (0)	1 (0)	
Preoperative glucose abnormalities, n (%)				0.04
Low	1386 (3)	1300 (3)	86 (3)	
Normal	35,027 (78)	32,475 (78)	2552 (80)	
High	8327 (19)	7778 (19)	549 (17)	
Preoperative white blood cell abnormalities, n (%)				0.41
Low	1280 (3)	1192 (3)	88 (3)	
Normal	37,868 (85)	35,191 (85)	2677 (84)	
High	5592 (12)	5170 (12)	422 (13)	
Preoperative hemoglobin abnormalities, n (%)				0.44
Low	15,903 (36)	14,737 (35)	1166 (37)	
Normal	28,605 (64)	26,600 (64)	2005 (63)	
High	232 (1)	216 (1)	16 (1)	
Preoperative routine glucocorticoid use, n (%)				0.06
No	44,645 (100)	41,470 (100)	3175 (100)	
Yes	95 (0)	83 (0)	12 (0)	
Preoperative routine anticoagulant use, n (%)				< 0.01
No	38,301 (86)	35,819 (86)	2482 (78)	
Yes	6439 (14)	5734 (14)	705 (22)	
Preoperative routine antidiabetic medication use, n (%)				0.04
No	40,057 (90)	37,238 (90)	2819 (88)	
Yes	4683 (10)	4315 (10)	368 (12)	
Preoperative routine antihypertensive medication use, n (%)				< 0.01
No	27,001 (60)	25,502 (61)	1499 (47)	
Yes	17,739 (40)	16,051 (39)	1688 (53)	
Preoperative statin use, n (%)				< 0.01
No	39,772 (89)	37,097 (89)	2675 (84)	
Yes	4968 (11)	4456 (11)	512 (16)	
Preoperative steroid use, n (%)				< 0.01
No	35,795 (80)	33,463 (81)	2332 (73)	
Yes	8945 (20)	8090 (19)	855 (27)	
History of anticoagulant Use, n (%)				0.02
No	44,490 (99)	41,331 (99)	3159 (99)	
Yes	250 (1)	222 (1)	28 (1)	

ASA, American Society of Anesthesiologists; ENT, ear, nose and throat.

**Table 2:** Baseline demographic and clinical characteristics of included patients between development cohort and validation cohort.

Variables	Total (n = 44,740)	Training cohort (n = 31,320)	Validation cohort (n = 13,420)	*P*
Postoperative pneumonia, n (%)				0.92
No	41,553 (93)	29,092 (93)	12,461 (93)	
Yes	3187 (7)	2228 (7)	959 (7)	
Sex, n (%)				0.18
Male	23,503 (53)	16,388 (52)	7115 (53)	
Female	21,237 (47)	14,932 (48)	6305 (47)	
Age, (median [Q1, Q3]), yr	70 (67, 74)	70 (67, 74)	70 (67, 75)	< 0.01
Height, median, (median [Q1, Q3]), cm	163.67 (157.5, 168.16)	163.53 (157.49, 168.16)	164 (157.52, 168.18)	0.34
Weight, (median [Q1, Q3]), kg	64.85 (56.5, 70)	64.19 (56.39, 70)	65 (57, 71)	0.28
ASA classification, n (%)				0.62
ASA I	518 (1)	353 (1)	165 (1)	
ASA II	31,872 (71)	22,331 (71)	9541 (71)	
ASA III	12,350 (28)	8636 (28)	3714 (28)	
Duration of surgery, (median [Q1, Q3]), minute	130 (75, 210)	130 (75, 209)	130 (75, 210)	0.67
Duration of anesthesia, (median [Q1, Q3]), minute	145 (85, 225)	145 (85, 225)	145 (85, 230)	0.61
Anesthesia Type, n (%)				0.9
Simple general anesthesia	31,468 (70)	22,023 (70)	9445 (70)	
General anesthesia combined with nerve block	13,272 (30)	9297 (30)	3975 (30)	
Emergency Surgery, n (%)				0.18
No	39,486 (88)	27,600 (88)	11,886 (89)	
Yes	5254 (12)	3720 (12)	1534 (11)	
Surgery type, n (%)				0.15
Abdominal_surgery	12,941 (29)	9140 (29)	3801 (28)	
ENT	2326 (5)	1663 (5)	663 (5)	
General_surgery	1826 (4)	1273 (4)	553 (4)	
Gynecologic_surgery	2110 (5)	1483 (5)	627 (5)	
Neurosurgery	2825 (6)	1920 (6)	905 (7)	
Ophthalmic_surgery	2910 (7)	2040 (7)	870 (6)	
Orthopedic_surgery	6445 (14)	4503 (14)	1942 (14)	
Other_surgeries	2828 (6)	1960 (6)	868 (6)	
Stomatology	1163 (3)	799 (3)	364 (3)	
Thoracic_surgery	1811 (4)	1239 (4)	572 (4)	
Urology	7555 (17)	5300 (17)	2255 (17)	
Smoking status, n (%)				0.57
No	34,002 (76)	23,827 (76)	10,175 (76)	
Yes	10,738 (24)	7493 (24)	3245 (24)	
Hypertension, n (%)				0.26
No	35,445 (79)	24,858 (79)	10,587 (79)	
Yes	9295 (21)	6462 (21)	2833 (21)	
Diabetes mellitus, n (%)				< 0.01
No	39,713 (89)	27,887 (89)	11,826 (88)	
Yes	5027 (11)	3433 (11)	1594 (12)	
Hyperlipidemia, n (%)				0.09
No	44,579 (100)	31,197 (100)	13,382 (100)	
Yes	161 (0)	123 (0)	38 (0)	
History of pulmonary disease, n (%)				0.51
No	43,774 (98)	30,634 (98)	13,140 (98)	
Yes	966 (2)	686 (2)	280 (2)	
Preoperative red blood cell abnormalities, n (%)				0.84
Low	17,432 (39)	12,213 (39)	5219 (39)	
Normal	27,019 (60)	18,909 (60)	8110 (60)	
High	289 (1)	198 (1)	91 (1)	
Preoperative lymphocyte abnormalities, n (%)				0.04
Low	8350 (19)	5781 (18)	2569 (19)	
Normal	35,492 (79)	24,884 (79)	10,608 (79)	
High	898 (2)	655 (2)	243 (2)	
Preoperative basophil abnormalities, n (%)				0.86
Normal	42,994 (96)	30,094 (96)	12,900 (96)	
High	1746 (4)	1226 (4)	520 (4)	
Preoperative platelet count abnormalities, n (%)				0.16
Low	2755 (6)	1949 (6)	806 (6)	
Normal	39,937 (89)	27,904 (89)	12,033 (90)	
High	2048 (5)	1467 (5)	581 (4)	
Preoperative total bilirubin, n (%)				0.14
Low	1713 (4)	1234 (4)	479 (4)	
Normal	38,990 (87)	27,244 (87)	11,746 (88)	
High	4037 (9)	2842 (9)	1195 (9)	
Preoperative albumin abnormalities, n (%)				0.12
Low	22,319 (50)	15,605 (50)	6714 (50)	
Normal	22,419 (50)	15,715 (50)	6704 (50)	
High	2 (0)	0 (0)	2 (0)	
Preoperative glucose abnormalities, n (%)				0.86
Low	1386 (3)	975 (3)	411 (3)	
Normal	35,027 (78)	24,534 (78)	10,493 (78)	
High	8327 (19)	5811 (19)	2516 (19)	
Preoperative white blood cell abnormalities, n (%)				0.21
Low	1280 (3)	921 (3)	359 (3)	
Normal	37,868 (85)	26,460 (84)	11,408 (85)	
High	5592 (12)	3939 (13)	1653 (12)	
Preoperative hemoglobin abnormalities, n (%)				0.74
Low	15,903 (36)	11,106 (35)	4797 (36)	
Normal	28,605 (64)	20,055 (64)	8550 (64)	
High	232 (1)	159 (1)	73 (1)	
Preoperative routine glucocorticoid use, n (%)				1
No	44,645 (100)	31,254 (100)	13,391 (100)	
Yes	95 (0)	66 (0)	29 (0)	
Preoperative routine anticoagulant use, n (%)				0.62
No	38,301 (86)	26,795 (86)	11,506 (86)	
Yes	6439 (14)	4525 (14)	1914 (14)	
Preoperative routine antidiabetic medication use, n (%)				0.23
No	40,057 (90)	28,078 (90)	11,979 (89)	
Yes	4683 (10)	3242 (10)	1441 (11)	
Preoperative routine antihypertensive medication use, n (%)				0.07
No	27,001 (60)	18,988 (61)	8013 (60)	
Yes	17,739 (40)	12,332 (39)	5407 (40)	
Preoperative statin use, n (%)				0.73
No	39,772 (89)	27,853 (89)	11,919 (89)	
Yes	4968 (11)	3467 (11)	1501 (11)	
Preoperative steroid use, n (%)				0.1
No	35,795 (80)	25,123 (80)	10,672 (80)	
Yes	8945 (20)	6197 (20)	2748 (20)	
History of anticoagulant use, n (%)				0.24
No	44,490 (99)	31,154 (99)	13,336 (99)	
Yes	250 (1)	166 (1)	84 (1)	

ASA, American Society of Anesthesiologists; ENT, ear, nose and throat.

Further analysis revealed significant variation in POP incidence across surgical categories (*P* < 0.001). Thoracic surgery had the highest rate (18.1%), followed by abdominal surgery (12.2%) and orthopedic surgery (6.4%). Moderate rates were observed in neurosurgery (4.4%), stomatology (4.6%), and urology (3.3%), while lower rates occurred in ENT (3.8%), gynecologic (3.7%), general (3.1%), and ophthalmic surgeries (1.5%).

Notably, while surgical type was not included as a predictor in the final model, these findings highlight its potential influence on POP risk. Thoracic and abdominal procedures, for example, may increase vulnerability to pulmonary complications due to their impact on respiratory mechanics, prolonged anesthesia requirements, and postoperative pain management. However, to preserve generalizability and avoid multicollinearity with other intraoperative variables (e.g., anesthesia duration, type), surgical type was excluded. Future models could benefit from stratified approaches or procedure-specific predictors to refine risk estimation.

### Variable selection and model construction

**Fig. 2:**
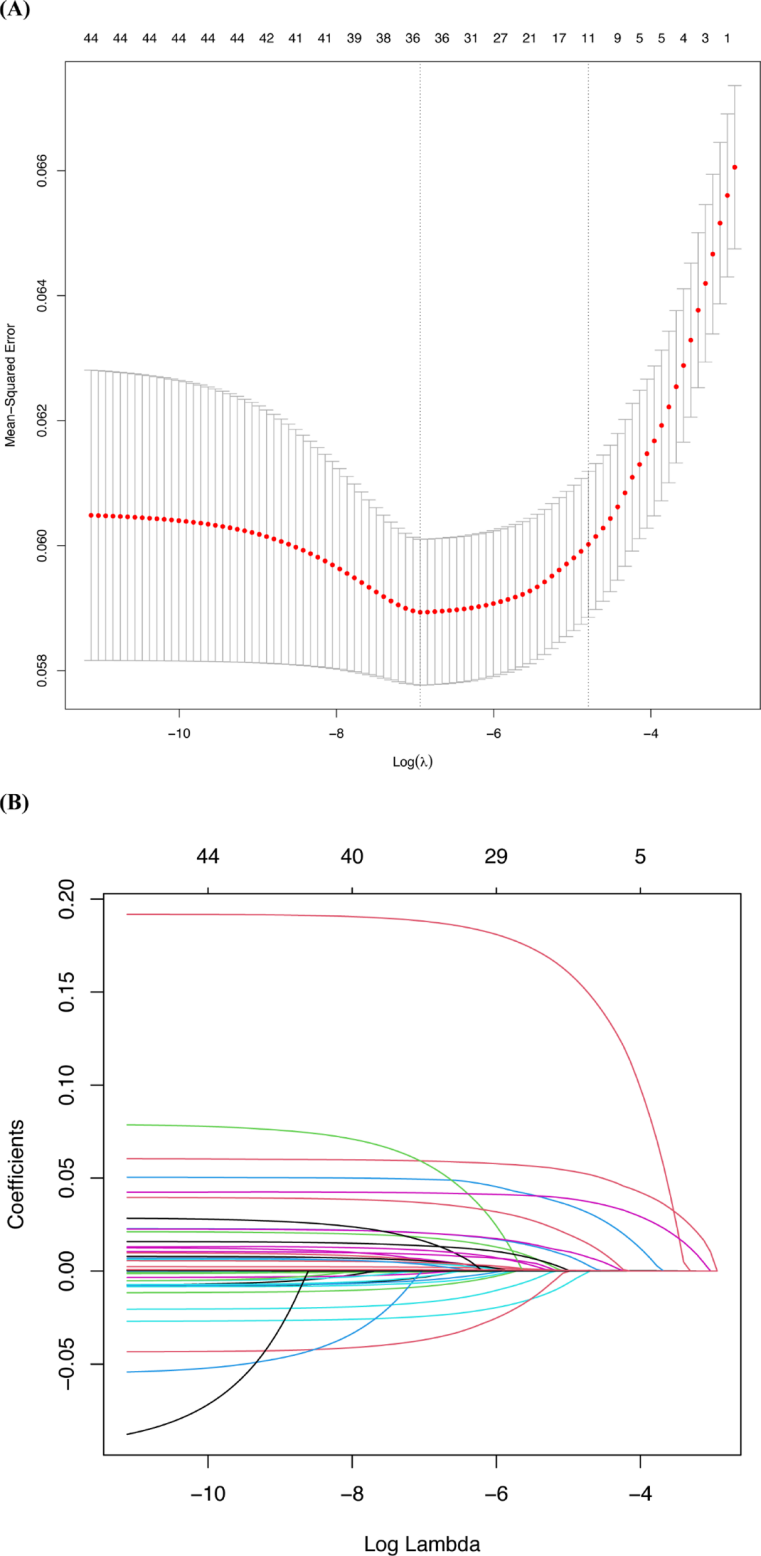
Factor selection using the least absolute shrinkage and selection operator (LASSO) logistic regression. (**A**) The LASSO coefficient profiles of the 44 candidate variables. A plot of the coefficient profile was generated against the log(λ). (**B**) Selection of the tuning parameter (λ) was performed using LASSO penalized logistic regression with 10 fold cross-validation.

To build the prediction model, LASSO logistic regression was first applied to 44 candidate variables. These variables were selected based on clinical relevance (supported by prior literature) and data availability in electronic medical records, capturing preoperative, intraoperative, and immediate postoperative characteristics linked to pulmonary complications. They included established risk factors (e.g., age, ASA class, smoking status, pulmonary comorbidities, anesthesia details) and variables with potential mechanistic ties to infection or respiratory function (e.g., laboratory values, medications). Using an optimal lambda value of 0.0083 (Fig. 2), nine variables with non-zero coefficients were retained.

**Fig. 3:**
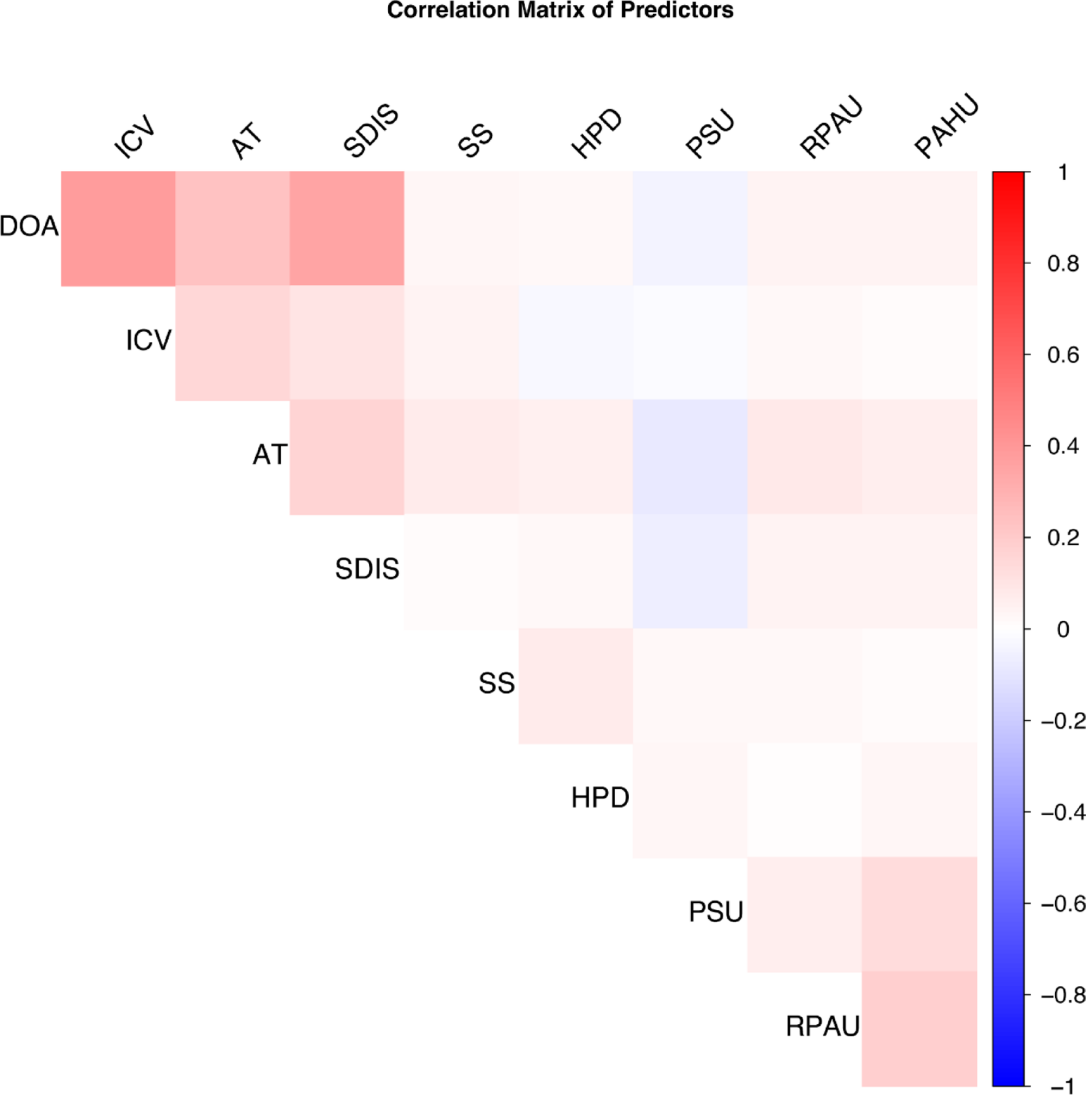
Heatmap of pairwise correlations among variables. This heatmap visualizes the pairwise correlations between the variables included in the model. Positive correlations are represented in red, while negative correlations are depicted in blue. The intensity of the color reflects the strength of the correlation, with darker shades indicating stronger correlations. DOA, duration of anesthesia; AT, anesthesia type; SS, smoking status; HPD, history of pulmonary disease; ICV, intraoperative colloid volume; RPAU, routine preoperative anticoagulant use; PAHU, routine preoperative antihypertensive use; PSU, preoperative steroid use; SDIS, single dose of intraoperative sufentanil.

These variables were entered into a multivariable logistic regression model, with forward stepwise selection (based on the Akaike Information Criterion [AIC]) to optimize the final model. The correlation matrix, variance inflation factors (VIF), and tolerance values for each variable are shown in Fig. 3 and Table 3, respectively.

**Table 3:** Variance inflation factors (VIF) and Corresponding tolerance values for each variable.

Variable	VIF	Tolerance
Duration of anesthesia	1.360928	0.7347929
Anesthesia type	1.097406	0.9112394
Smoking status	1.013263	0.9869104
History of pulmonary disease	1.011783	0.9883547
Intraoperative colloid volume	1.186654	0.8427059
Routine preoperative anticoagulant use	1.043449	0.9583604
Routine preoperative antihypertensive use	1.058225	0.9449783
Preoperative steroid use	1.037075	0.9642505
Single dose of intraoperative sufentanil	1.154415	0.8662397

The resulting nine predictors included:

1. Duration of anaesthesia (OR: 1.01, 95% CI 1.01–1.01)
2. Type of anaesthesia (OR: 3.84, 95% CI 3.49–4.23)
3. Smoking status (OR: 1.92, 95% CI 1.75–2.12)
4. History of pulmonary disease (OR: 4.38, 95% CI 3.63–5.29)
5. Intraoperative colloid volume (OR: 1.00, 95% CI 1.00–1.00)
6. Routine preoperative anticoagulant use (OR: 1.42, 95% CI 1.27–1.59)
7. Routine preoperative antihypertensive use (OR: 1.38, 95% CI 1.26–1.52)
8. Preoperative steroid use (OR: 1.61, 95% CI 1.44–1.80)
9. Intraoperative single dose of sufentanil (OR: 1.01, 95% CI 1.01–1.01)

Detailed coefficients and *p*-values are presented in Table 4.

**Table 4:** Multivariate logistic regression analysis for risk factors associated with postoperative pneumonia in perioperative non-cardiac surgery patients.

	B	SE	OR	CI	Z	*P*
Duration of anesthesia	0.005	0	1.01	1.01–1.01	23.747	0
Anesthesia type [pure general anesthesia]	1.346	0.049	3.84	3.49–4.23	27.414	0
Smoking status [yes]	0.654	0.049	1.92	1.75–2.12	13.351	0
History of pulmonary disease [yes]	1.478	0.096	4.38	3.63–5.29	15.364	0
Intraoperative colloid volume	− 0.001	0	1	1.00–1.00	− 14.37	0
Routine preoperative anticoagulant use [yes]	0.351	0.058	1.42	1.27–1.59	6.017	0
Routine preoperative antihypertensive use [yes]	0.326	0.049	1.38	1.26–1.52	6.701	0
Preoperative steroid use [yes]	0.476	0.056	1.61	1.44–1.8	8.448	0
Single dose of intraoperative sufentanil	0.01	0.002	1.01	1.01–1.01	5.583	0

B, regression coefficient; SE, standard error; OR, odds radio; CI, credibility interval.

### Model performance and internal validation

**Fig. 4:**
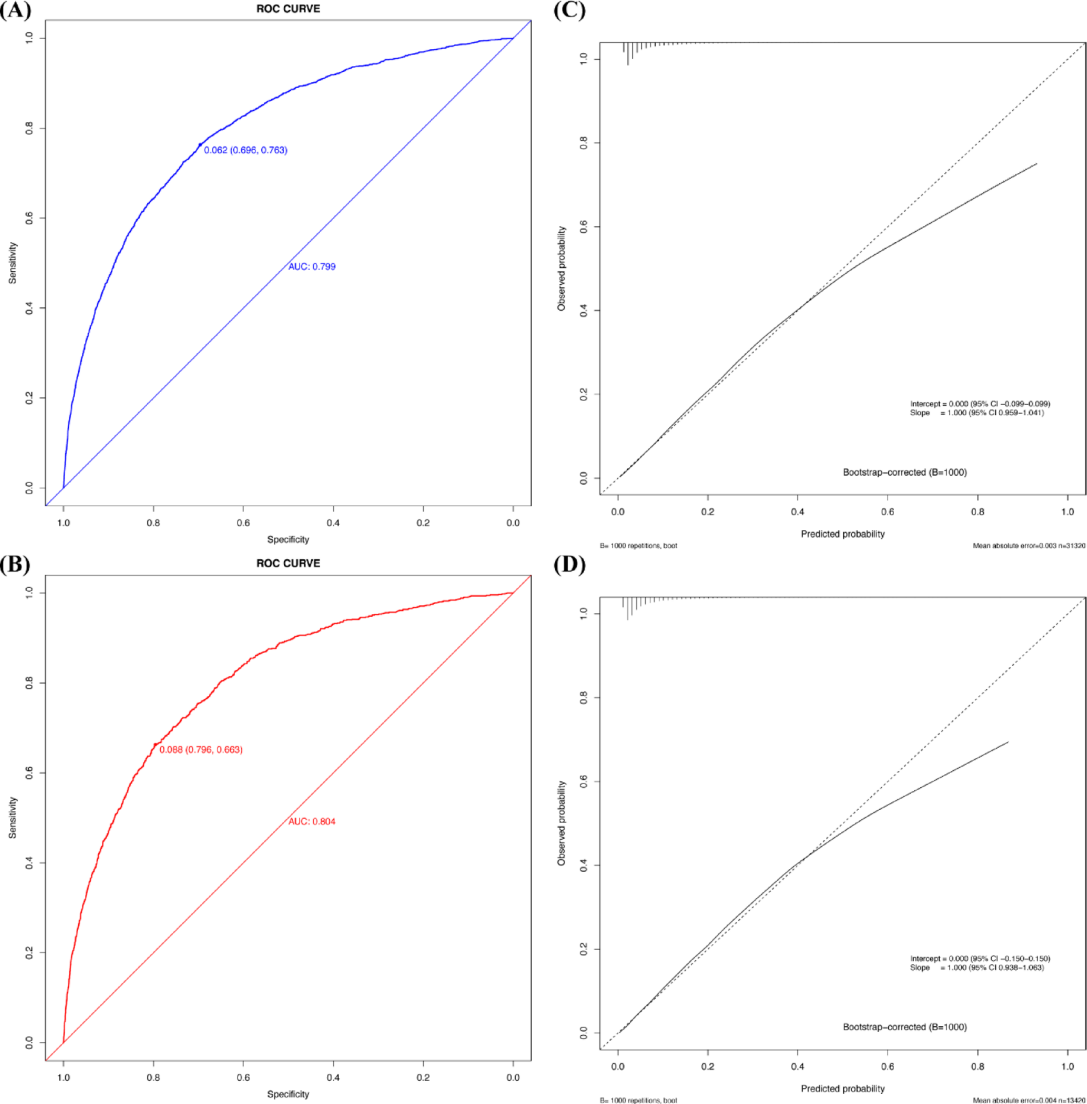
The ROC curve, calibration plot of the new predictive model. (**A**) ROC curve of the new predictive model with the development cohort; (**B**) ROC curve for the new prediction model with the validation cohort; (**C**) Calibration plot of the new predictive model with the development cohort; (**D**) Calibration plot of the new predictive model with the validation cohort.

The model showed strong discriminatory performance. In the development cohort, the area under the curve (AUC) was 0.799 (95% CI 0.789–0.808) with a C-index of 0.799. In the validation cohort, the AUC was 0.804 (95% CI 0.790–0.818) with a C-index of 0.804 (Figs. 4A, B). This exceeds conventional benchmarks for clinical prediction models, indicating excellent discriminative ability. For postoperative pneumonia risk stratification—where early identification of high-risk patients is critical—an AUC > 0.8 suggests clinically meaningful discrimination that may support real-world decision-making.

### Calibration and goodness-of-fit

Calibration was evaluated using both visual and quantitative methods. The Brier score, reflecting overall accuracy of probabilistic predictions, was 0.058 in both the development and validation cohorts. The Hosmer–Lemeshow goodness-of-fit test yielded χ^2^ = 5.45 (*P* = 0.79) for the development cohort and χ^2^ = 7.81 (*P* = 0.55) for the validation cohort, indicating no significant discrepancy between predicted and observed outcomes. These results confirm satisfactory calibration.

Calibration curves following bootstrap optimism correction (1000 resamples) are shown in Figs. 4C, D. They demonstrate strong concordance between predicted and actual probabilities across the risk spectrum. The corrected intercept and slope (annotated on the plots) further support reliable calibration and minimal overfitting.

### Clinical utility: decision curve analysis and nomogram

**Fig. 5:**
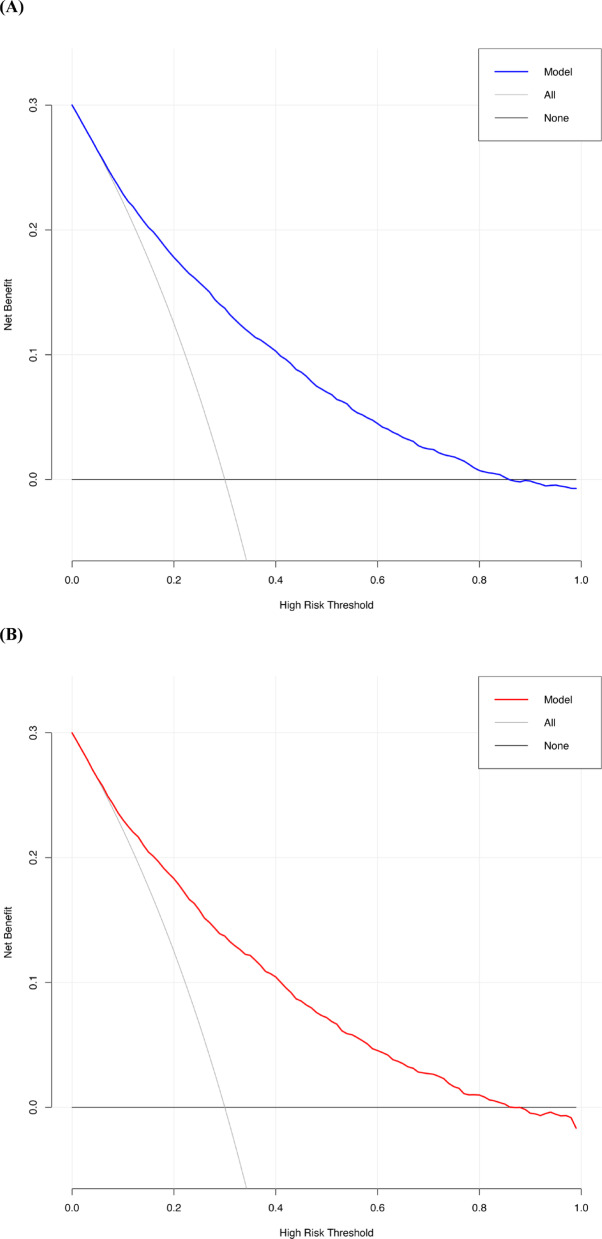
The decision curve of the new predictive model. (**A**) Decision curve of the new predictive model with the development cohort; (**B**) Decision curve of the new predictive model with the validation cohort.

Decision curve analysis (DCA) was used to evaluate clinical net benefit. The model showed positive net benefit across a wide range of threshold probabilities: 0–89% in the development cohort and 0–88% in the validation cohort (Figs. 5A, B), supporting its utility for perioperative risk stratification in elderly noncardiac surgical patients.

**Fig. 6:**
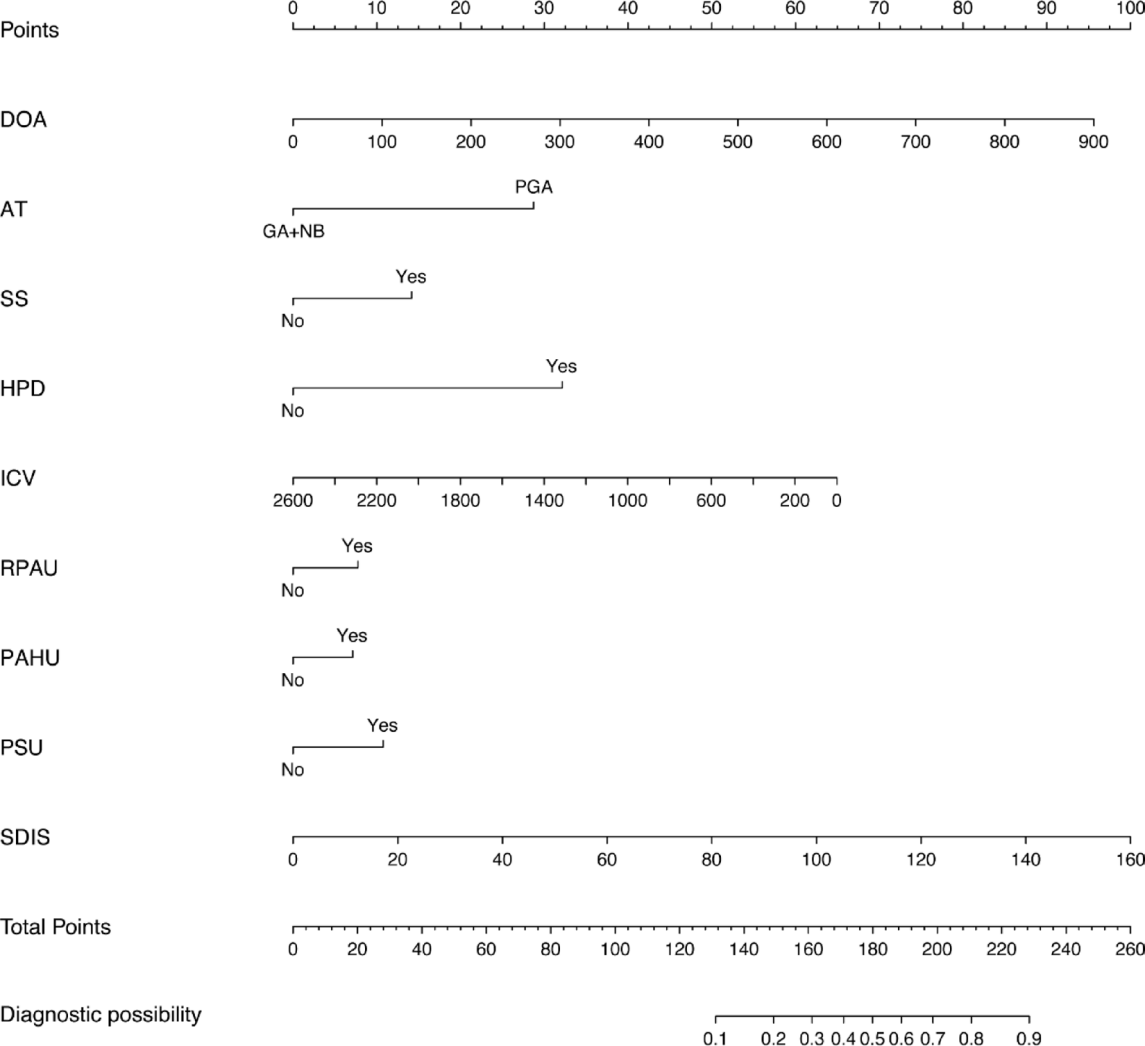
Nomogram of the logistic regression model for perioperative POP risk prediction. DOA, duration of anesthesia; AT, anesthesia type; SS, smoking status; HPD, history of pulmonary disease; ICV, intraoperative colloid volume; RPAU, routine preoperative anticoagulant use; PAHU, routine preoperative antihypertensive use; PSU, preoperative steroid use; SDIS, single dose of intraoperative sufentanil; GA + AB, general anesthesia combined with nerve block; PGA: pure general anesthesia.

Figure 6 presents a nomogram illustrating the logistic model. Anesthesia duration, intraoperative colloid volume, and intraoperative single dose of sufentanil are treated as continuous variables; the remaining variables are categorical. Using a Youden index of 0.60, the optimal cutoff value on the nomogram was 190, yielding high sensitivity (76.3%) and specificity (69.6%). Scores > 190 indicate high perioperative POP risk.

### Model interpretability and visualization

**Fig. 7:**
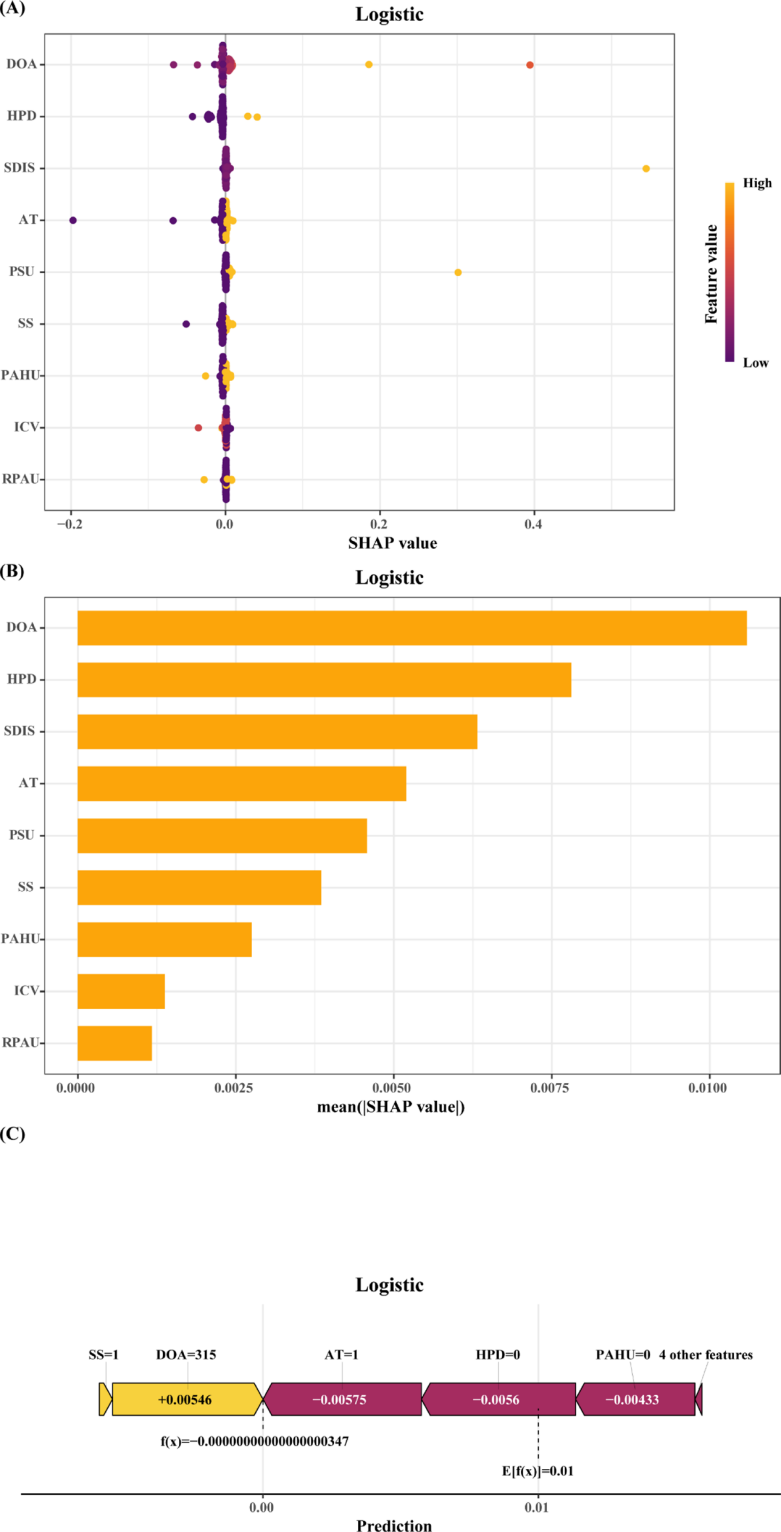
SHAP of the model: (**A**) Characteristic attributes in SHAP. The abscissa is the SHAP value, and each line denotes a feature. Higher eigenvalues are indicated by purple dots, and lower eigenvalues are indicated by yellow dots; (**B**) Feature importance ranking of the logistic regression model; (**C**) Interpretability analysis of 1 independent samples. DOA, duration of anesthesia; AT, anesthesia type; SS, smoking status; HPD, history of pulmonary disease; ICV, intraoperative colloid volume; RPAU, routine preoperative anticoagulant use; PAHU, routine preoperative antihypertensive use; PSU, preoperative steroid use; SDIS, single dose of intraoperative sufentanil.

Figure 7A shows the SHAP (SHapley Additive exPlanations) summary plot for the logistic regression model. The x-axis denotes SHAP values (reflecting each feature’s contribution to predicted POP risk), with color gradients (purple for high values, yellow for low) representing feature levels.

The four most influential predictors of POP were prolonged anesthesia duration, history of pulmonary disease, higher intraoperative single dose of sufentanil, and use of general anesthesia alone—all strongly associated with increased risk. Other key contributors included absence of preoperative steroid use, non-smoking history, lower frequency of routine preoperative antihypertensive and anticoagulant use, and appropriate intraoperative colloid administration.

Figure 7B confirms these findings by ranking features by their relative importance in the model. Figure 7C further illustrates interpretability via a SHAP force plot for a representative non-POP case, visualizing individualized feature contributions to predicted risk.

**Fig. 8:**
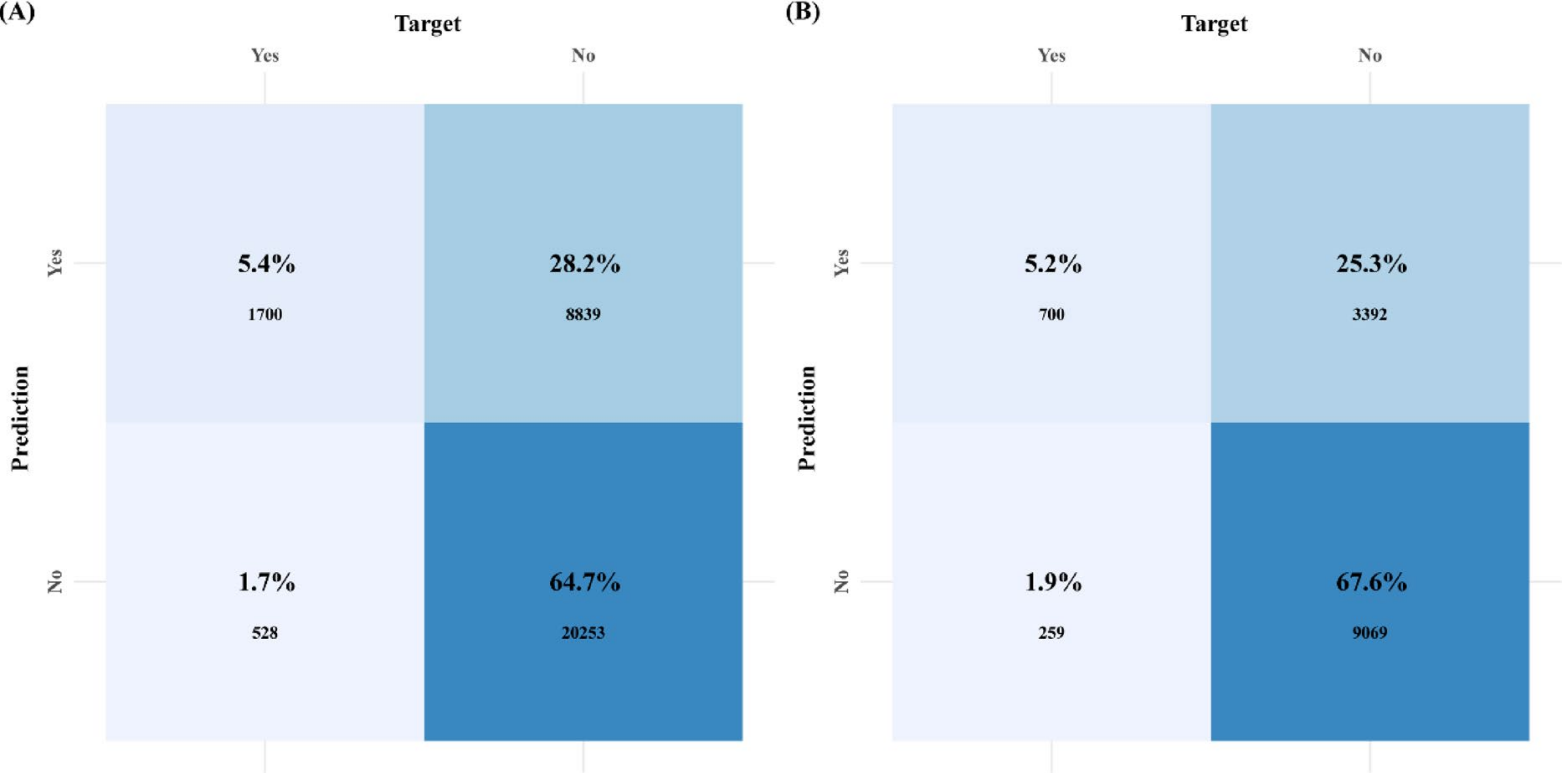
Confusion matrix for the model: (**A**) Confusion matrix for the development cohort; (**B**) Confusion matrix for the validation cohort.

Complementing these analyses, Figs. 8A, B present the model’s confusion matrices, comparing actual versus predicted classifications to clarify diagnostic performance.

## Discussion

Postoperative pneumonia (POP) is a common and serious perioperative complication that significantly impairs patient recovery and long-term prognosis. Its development is influenced by multiple factors, including underlying comorbidities, anesthesia type, intraoperative procedures, and medication use^11,12^. Numerous studies have identified perioperative risk factors—such as advanced age, prolonged surgery duration, general anesthesia, smoking history, and pulmonary disease history—as strongly associated with increased POP risk^13–15^. Detailed analysis of these factors not only enhances understanding of POP pathogenesis but also provides a foundation for developing risk prediction models to guide individualized perioperative interventions, thereby reducing complication rates^16^.

The clinical relevance of specific POP risk factors may vary by surgical category. In our analysis, thoracic and abdominal surgeries had the highest POP incidence (18.1% and 12.2%, respectively), likely due to impaired respiratory mechanics, extended anesthesia duration, and heightened postoperative pain. These findings align with evidence that upper abdominal and thoracic procedures increase pulmonary complication risk by reducing diaphragmatic excursion and disrupting ventilation-perfusion matching^17,18^. Orthopedic surgeries showed moderate POP rates (6.4%), potentially linked to postoperative immobility and opioid-induced hypoventilation^19^, while less invasive procedures (e.g., gynecologic, ENT) had lower rates. These trends support surgical invasiveness as a key modulator of POP risk^20^.

Notably, although surgical type was excluded from our final model to avoid multicollinearity with intraoperative variables (e.g., anesthesia duration and type), the observed intergroup differences suggest that stratified modeling could enhance risk prediction in future studies.

In this study, we developed a POP risk prediction model using key perioperative factors, including anesthesia type, anesthesia duration, smoking history, pulmonary disease history, preoperative medication use, and intraoperative medication management. Results highlighted anesthesia type and duration as critical contributors to POP risk^21^. Compared with local or regional anesthesia, general anesthesia increases POP risk, potentially by impairing airway defenses via intubation and mechanical ventilation (promoting pathogen colonization)^15^ and suppressing respiratory function (reducing postoperative lung clearance)^13^. Prolonged anesthesia exacerbates this risk, consistent with studies linking extended mechanical ventilation to impaired lung defenses and higher POP incidence^14^.

Smoking and pulmonary disease history are also significant POP risk factors. Smoking damages lung structures, weakens immune function, and impairs pathogen clearance, increasing susceptibility to postoperative infections^16,22^. Patients with preexisting pulmonary conditions (e.g., COPD, asthma) have lower tolerance for surgery and anesthesia, making them more prone to postoperative pulmonary complications^21^. These findings underscore the value of preoperative pulmonary function assessments and smoking cessation interventions for high-risk patients^12^.

Preoperative medications—including anticoagulants, antihypertensives, steroids, and statins—also influence POP risk. Anticoagulants may increase bleeding risk, indirectly impairing immune responses, while long-term steroid use can suppress immunity and enhance infection susceptibility^23,24^. Conversely, preoperative statin use has anti-inflammatory effects that may protect against postoperative complications^25^. Future research should explore these mechanisms to better quantify medication impacts on POP.

Intraoperative factors also play a role: higher single doses of sufentanil were associated with increased POP risk, likely due to opioid-induced respiratory depression (impairing airway clearance and promoting infections)^26^. This highlights the need to balance analgesia with respiratory function during the perioperative period. Additionally, appropriate intraoperative colloid administration may reduce POP risk, potentially by optimizing intravascular volume and tissue perfusion. This aligns with studies showing that goal-directed fluid therapy—especially balanced colloid use—lowers postoperative pulmonary complication rates^27,28^; incorporating refined fluid metrics could further improve model performance.

Several risk factors showed modest but clinically meaningful associations with POP. For example, routine preoperative anticoagulant use was linked to a 42% increased odds of POP (OR = 1.42, 95% CI 1.27–1.59). While the effect size is small, its relevance lies in the high prevalence of anticoagulant use among elderly surgical patients (38.2% in our cohort) and cumulative risk when combined with other factors (e.g., prolonged anesthesia, pulmonary disease history)^29^. This suggests preoperative assessment of anticoagulant therapy—including temporary discontinuation or bridging in low-thromboembolic-risk patients—may reduce POP risk without compromising thromboprophylaxis.

Similarly, preoperative antihypertensive use (OR = 1.38, 95% CI 1.26–1.52) likely reflects underlying cardiovascular comorbidity, a marker of frailty in elderly patients. Even a modest OR signals increased vulnerability to perioperative stress, emphasizing the need for optimized blood pressure control and hemodynamic monitoring^30^.

For continuous variables like anesthesia duration (OR = 1.01, 95% CI 1.01–1.01 per minute), the OR reflects incremental risk per minute. Clinically, a 60-min increase (common between intermediate and complex surgeries) corresponds to a ~ 70% higher POP odds (OR = 1.01^60≈1.82), while a 30-min prolongation (e.g., unplanned complexity) increases odds by ~ 35% (1.01^30≈1.35). These examples show small per-minute effects accumulate substantially, reinforcing the importance of optimizing surgical efficiency in high-risk patients^31,32^.

In summary, we developed a multifactorial POP prediction model for elderly noncardiac surgical patients. Integrating routinely available perioperative variables, the model showed strong internal performance and may facilitate early identification of high-risk individuals, supporting personalized perioperative strategies to reduce POP risk.

Notably, our model advances prior work in several key aspects. A study by Wang et al.^33^ developed a POP prediction model specifically for lung cancer surgery patients, with a cohort of 1252 cases. Their model included six predictors (smoking, diabetes mellitus, history of preoperative chemotherapy, thoracotomy, ASA grade, and surgery time) and yielded relatively lower AUC values (0.717 in the training cohort and 0.726 in the validation cohort). However, this study was limited to a single surgical subtype (lung cancer resection) and did not incorporate intraoperative variables such as anesthesia type or analgesic medication doses, which our analysis identifies as critical predictors of POP.

Another relevant study by Liu et al.^34^, focusing on surgical outcomes after total knee arthroplasty (a representative orthopedic procedure), highlighted the application of machine learning in perioperative risk prediction but was restricted to a single surgical specialty. Similar to Wang et al.’s work, its predictive framework did not include intraoperative factors like anesthesia duration or colloid administration—variables that our model demonstrates to be clinically meaningful for POP risk stratification.

In contrast, our model benefits from a substantially larger and more diverse cohort (44,740 elderly patients undergoing noncardiac surgery across multiple specialties), incorporates variables spanning preoperative, intraoperative, and immediate postoperative phases (e.g., anesthesia type, intraoperative sufentanil dose), and achieves higher discriminative performance (validation AUC = 0.804). Additionally, the integration of SHAP analysis enhances interpretability by clarifying the contribution of each predictor, addressing a common limitation of prior models that lack transparent feature relevance^33,34^. These improvements strengthen the clinical applicability of our model for broader elderly noncardiac surgical populations.

This study has several limitations. First, as a single-center analysis, the findings may not be generalizable to other institutions with different patient populations, surgical practices, or perioperative protocols^35^. Second, the retrospective design introduces potential selection bias and unmeasured confounding^36^. Additional bias may arise from institutional documentation variability or underreporting of comorbidities and medications, as the data were extracted from a single-center HIS-based perioperative database. To mitigate these issues, we applied standardized inclusion criteria, used multiple imputation for missing data, and followed a rigorous data cleaning protocol. Nonetheless, inherent limitations of retrospective EMR-based studies remain and highlight the need for external validation.

Third, defining POP as occurring within 48 h postoperatively—intended to capture early-onset events attributable to intraoperative care—may underestimate later-onset but clinically relevant pneumonia cases. Fourth, our model was not compared with existing risk prediction tools, limiting contextual interpretation of its performance. Fifth, real-time intraoperative data and geriatric-specific indicators (e.g., frailty, polypharmacy) were not included, which may reduce the model’s comprehensiveness. Finally, the model has not yet undergone external validation, and its clinical utility remains to be confirmed in prospective, multicenter cohorts.

Future studies should validate this model using multicenter datasets, incorporate additional perioperative variables (e.g., early postoperative lung function rehabilitation and real-time respiratory monitoring), and include geriatric-specific factors to improve prediction accuracy and applicability.

## Conclusion

This study developed a multifactorial model to predict postoperative pneumonia (POP) in elderly noncardiac surgical patients using routinely collected perioperative variables. The model demonstrated good discrimination and calibration in both the development and internal validation cohorts, supporting its potential for early identification of high-risk individuals.

By incorporating a broader range of perioperative factors than previous models, this study improves both predictive accuracy and clinical utility. Future research should focus on validating the model in prospective, multicenter cohorts to facilitate its integration into perioperative decision-making and improve outcomes for elderly surgical patients.

## Data Availability

The analytical code, minimal datasets, and supporting documentation used in this study are publicly available at our GitHub repository: https://github.com/Iory-lab/POP-Model-1.0. The repository includes the training set, validation set, and the complete minimal dataset used for model development and internal validation. The analytical code is archived under commit 9b2be03, and the documentation (README) under commit 5f64550. Due to patient privacy regulations, the full electronic medical records cannot be shared. However, de-identified data sufficient to reproduce the study findings are available within the repository. Further inquiries can be directed to the corresponding author.
